# Tumor Infiltrating Lymphocytes – The Next Step in Assessing Outcome and Response to Treatment in Patients with Breast Cancer

**DOI:** 10.4172/2157-2518.1000199

**Published:** 2014-11-25

**Authors:** Robert Wesolowski, William E Carson

**Affiliations:** 1 Center of Internal Medicine, Division of Medical Oncology, Ohio State University, Columbus, USA; 2 Department of Surgical Oncology; OSU Comprehensive Cancer Center, Ohio State University, Columbus, USA

**Keywords:** Breast cancer, Tumor infiltrating lymphocytes, Biomarkers

## Abstract

Tumor infiltrating lymphocytes are studied for their potential as new clinically useful prognostic and predictive biomarkers in patients with triple negative and HER-2/neu amplified breast cancer. This area of research could also help guide the development of novel therapeutic approaches for these diseases.

## Introduction

Over the recent years, new results from multiple groups have pointed towards Tumor Infiltrating Lymphocytes (TILs) as prognostic and predictive biomarkers in breast cancer. This is not a new concept. For instance, it has been known since 1940s that a subtype of breast cancer that characteristically exhibits a very large proportion of stromal lymphocytic infiltrate, called medullary carcinoma, has been associated with excellent prognosis after aggressive local therapy in spite of high histologic grade and axillary lymph node metastases [1,2]. However, over the next several decades, a number of studies examined the association between tumor lymphocytic infiltrates and prognosis in more common histologic types of breast cancer and yielded contradictory results [3–6]. The concept of the immune tumor microenvironment’s role in influencing the outcomes of breast cancer patients has resurfaced after the recognition that expression of genes associated with stromal (fibroblast) and immune (macrophage, T-cell) components significantly contributes to the global gene expression landscape within the breast tumors as detected by the use of cDNA based gene expression microarrays [7]. High expression of genes typical for lymphocytes (such as CD8) and low expression of genes typical of myeloid cells (such as CD68) was found to indicate better prognosis, especially in basal like and HER-2/neu positive breast cancer subtypes [8–10]. This has generated a renewed excitement and multiple investigators began to study the potential association between TILs, prognosis and response to systemic therapy for breast cancer. Lymphocytes can be easily detected by analyzing slides of tumor sections stained with hematoxylin and eosin (H&E); a simple and inexpensive technique that can be reliably performed by practically any pathologist.

## Tumor Infiltrating Lymphocytes and Adjuvant Chemotherapy Trials

To study the association between TILs (detected on H&E stain) and survival, investigators utilized tumors collected from breast cancer patients who participated in randomized clinical trials of adjuvant chemotherapy. The advantage of this approach is the availability of samples from large cohorts of patients with abundance of data on clinical characteristics of their disease and their outcomes. By using such an approach, Loi and colleagues have demonstrated that a high proportion of TILs (>50%) in tumors of women with operable, triple negative breast cancer enrolled in a large adjuvant chemotherapy trial called BIG 2-98, was strongly associated with favorable disease free (p=0.014) and overall (p=0.029) survival [11]. In their analysis, two distinct subsets of lymphocytes were measured, namely (1) stromal TILs (sTILs – mononuclear cells that were present within the tumor stroma but were not in direct contact with invasive carcinoma cells) and (2) intratumoral TILs (iTILs – mononuclear cells that were directly associated with the malignant cells). Their analysis revealed that for every 10% increase in the levels of sTILs there was 17% reduction in the risk of relapse (p=0.025) and death (p=0.023). They also found close association between higher levels of sTILs and iTILs with infiltrating ductal histology (P<0.001 and 0.48 respectively), high histologic grade (both p<0.001), hormone receptor negativity (both p<0.001), and increased Ki67 expression (>14%; both p<0.001). These results suggest that perhaps lymphocyte predominance could select a subgroup of patients with favorable prognosis despite having other poor clinical and histologic characteristics. No association was found between TIL levels and outcomes of patients with hormone receptor positive or HER-2/neu amplified breast cancer. In the recently presented confirmatory study, tumor samples of women with operable triple negative breast cancer from two large adjuvant trials (ECOG 2197 and ECOG 1199) were analyzed for the presence of TILs [12]. Again, the investigators found that for every 10% increase in sTILs there was 18% reduction in the risk of distant recurrence and 19% reduction in the risk of death. The presence of iTILs showed a trend towards better outcome but it did not reach statistical significance. On multivariate analysis, high levels of sTILs predicted improved disease free, distant recurrence free and overall survival independently of other poor clinical and histologic characteristics such as tumor size, lymph node metastases or patient age. Similar findings were shown in patients with triple negative and HER-2/neu positive breast cancer enrolled on a phase III, randomized FinHER trial that tested different adjuvant chemotherapy regiments with or without trastuzumab. This analysis also suggested for the first time that higher levels of TILs could be associated with increased trastuzumab benefit in HER-2/neu positive breast cancer [13].

## Tumor Infiltrating Lymphocytes and Neo-adjuvant Chemotherapy Trials

Other studies looked at association between levels of TILs and the likelihood of achieving a complete pathologic response (pCR) following neo-adjuvant chemotherapy for breast cancer. Complete pathologic response is a surrogate biomarker that is generally considered to indicate a lower likelihood of recurrence and a higher chance of survival [14]. Loi and colleagues analyzed biopsy specimen from 156 patients with HER-2/neu positive breast cancer enrolled in GeparQuatro trial and treated with trastuzumab containing neoadjuvant chemotherapy [15]. In this study, every 10% increase in the levels of TILs resulted in 16% increase in the likelihood of achieving pCR. Forty seven percent of patients with high levels of TILs achieved pCR in contrast to only 31.7% of patients in the entire cohort. This provided additional support to the notion that TIL levels might correlate with the response to trastuzumab based chemotherapy. In another analysis of tumor samples from patients enrolled in a neoadjuvant Neo-Sphere trial that tested neo-adjuvant pertuzumab, trastuzumab, or both, with or without chemotherapy, Gianni and colleagues discovered that high expression of immune checkpoints within breast tumors, such as PD1 and PDL1 (which inhibit the function of T-lymphocytes) were associated with lower likelihood of achieving pCR after chemotherapy in combination with trastuzumab and pertuzumab [16]. Other groups have found a correlation between levels of TILs and response to carboplatin. In an analysis of 580 diagnostic tumor biopsies obtained from patients on a clinical trial testing neo-adjuvant chemotherapy with or without carboplatin (Gepar Sixto trial), approximately 60% of patients with lymphocyte predominant breast cancer (defined as presence of ≥60% of TILs) achieved pCR compared to pCR rate of 40% in all participants and 34% in patients with low levels of TILs (p<0.0005) [17]. Among patients with high levels of TILs, those who received carboplatin achieved a pCR rate of 74% compared to 46.6% in patients treated without carboplatin. A similar study by Vinayak and colleagues analyzed diagnostic biopsies of patients with triple negative breast cancer treated with neo-adjuvant carboplatin, gemcitabine and a Poly (ADP – Ribose) Polymerase (PARP) inhibitor iniparib [18]. In this analysis, tumors with high levels of TILs were tightly correlated with an immunomodulatory subtype of triple negative breast cancer, based on Vanderbilt classification [19]. There was a strong association between high levels of both sTIL and iTIL and residual tumor burden score (an objective method of assessing pathologic response to neoadjuvant chemotherapy). However, an association remained statistically significant only for iTILs after adjustments for age, tumor size, N stage, tumor grade and germ line BRCA status. For every 10% increase in iTILs there was a 162% increase in the odds of achieving pCR.

## Clinical Significance and Future Directions

What are the implications of these findings to current clinical practice? Perhaps, the results of the above studies could lead to the routine practice of reporting the levels of lymphocytic infiltrates in the pathology reports of patients diagnosed with breast carcinoma (especially in cases of triple negative and HER-2/neu positive disease)? Before that occurs, the methodology of interpreting TILs and the cut off values for lymphocyte predominant breast cancer will need to be standardized. This data will also require future validation by other groups in prospectively designed clinical trials with pre-specified immune correlative endpoints. Regardless, the findings from the above analysis are already providing crucial clues for future clinical research. As an example, these studies corroborate pre-clinical work by others suggesting that perhaps some conventional anti-neoplastic drugs such as platinum agents or trastuzumab could induce anti-tumor responses at least in part by modulating the host immune system [13,17,20–23]. The finding that high expression of immune checkpoints is associated with lower pCR rates after neo-adjuvant therapy with monoclonal antibodies against HER-2/neu, provides support for studying combinations of trastuzumab and/or pertuzumab and PD1, PDL1 or CTLA-4 inhibitors. Additionally, since high levels of TILs are most common in the immune-modulatory subtype of triple negative breast cancer, it may be reasonable to select such patients for treatment with immune based cancer therapies. Another important issue that is currently being studied is whether predominance of different subtypes of intra-tumoral lymphocytes (such as CD4+ T-helper cells, CD8+ cytotoxic T-lymphocytes or CD25+/FOXP3+ T-regulatory cells) could have differential effect on patient outcome. This requires more complex analyses using immunohistochemical stains, fluorescent microscopy, flow cytometry or gene expression microarrays. One such method called Cibersort is a gene expression based assay that can identify 23 leukocyte subsets present in the tumor based on a unique gene expression signature of these cells. By using Cibersort, Vinayak et al. demonstrated that high levels of activated CD4+ memory T-cells were significantly associated with pathologic response to neo-adjuvant carboplatin containing chemotherapy [18]. One more critical question is why some breast cancer patients have high levels of TILs while others don’t. Understanding this may provide clues for new effective therapeutic strategies. In conclusion, the work summarized above could, in the near future, lead us towards developing vital prognostic and predictive biomarkers that would guide the selection of the most effective management strategies for breast cancer and provide support for the development of novel treatments [24].
